# Dynamic Network Analysis of COVID-19 with a Latent Pandemic Space Model

**DOI:** 10.3390/ijerph18063195

**Published:** 2021-03-19

**Authors:** Amanda M. Y. Chu, Thomas W. C. Chan, Mike K. P. So, Wing-Keung Wong

**Affiliations:** 1 Department of Social Sciences, The Education University of Hong Kong, Tai Po, Hong Kong; amandachu@eduhk.hk; 2 Department of Information Systems, Business Statistics and Operations Management, The Hong Kong University of Science and Technology, Clear Water Bay, Hong Kong; imthomas@ust.hk (T.W.C.C.); immkpso@ust.hk (M.K.P.S.); 3 Department of Finance, Fintech & Blockchain Research Center, and Big Data Research Center, Asia University, Taichung 41354, Taiwan; 4 Department of Medical Research, China Medical University Hospital, Taichung 404, Taiwan; 5 Department of Economics and Finance, The Hang Seng University of Hong Kong, Shatin, Hong Kong

**Keywords:** coronavirus, network modeling, pandemic nowcasting, pandemic risk visualization, pandemic network analysis, pandemic space

## Abstract

In this paper, we propose a latent pandemic space modeling approach for analyzing coronavirus disease 2019 (COVID-19) pandemic data. We developed a pandemic space concept that locates different regions so that their connections can be quantified according to the distances between them. A main feature of the pandemic space is to allow visualization of the pandemic status over time through the connectedness between regions. We applied the latent pandemic space model to dynamic pandemic networks constructed using data of confirmed cases of COVID-19 in 164 countries. We observed the ways in which pandemic risk evolves by tracing changes in the locations of countries within the pandemic space. Empirical results gained through this pandemic space analysis can be used to quantify the effectiveness of lockdowns, travel restrictions, and other measures in regard to reducing transmission risk across countries.

## 1. Introduction

Since early 2020, many countries have been affected by the novel coronavirus disease 2019 (COVID-19) pandemic. Large numbers of confirmed cases of COVID-19 have been reported in the time since the World Health Organization’s (WHO) declaration of COVID-19 as a global pandemic on 11 March 2020 [1]. By 22 July 2020, there had been 14,780,939 confirmed cases and 608,839 deaths [2]. There is no doubt that the outbreak of COVID-19 has resulted in a serious threat to public health. To stop the spread of the disease and to reduce the risk from the pandemic, different countries have adopted various levels of control measures, including, but not limited to, quarantining, social distancing regulations, travel restrictions, and the locking down of entire cities.

There has been growing interest in research into various aspects of the COVID-19 pandemic, including the ways in which the disease is spreading [3,4,5]. There is also a lot of ongoing research into the various psychological [6,7], environmental [8,9], economic [10,11], and financial [12,13] impacts of COVID-19 and the policies that have been adopted in response to these. There have been several studies carried out to evaluate the effectiveness and impacts of social distancing measures [14]. The findings of these studies have shown that lockdown measures, in particular, appear to be one of the most effective approaches to limiting the spread of infections [15]. Studies have also shown that the imposition of travel restrictions has been effective in reducing correlations in the numbers of infected people across different countries [16,17]. In this paper, we develop a dynamic pandemic network model to evaluate the performances of several major policies in terms of controlling the spread of COVID-19. The policies include travel restrictions, lockdowns, and reopenings, and the adoption of these policies in different countries is investigated using a pandemic space concept. The pandemic space considered here is not an absolute space, which defines a coordinate system with objects inside the space linked under the governance of Euclidean geometry [18]. Rather, the pandemic space refers to the relationship between objects under the context of interest [19]. In our case, to have a numerical representation of similarities in the prevalence of the pandemic between countries, we insert a coordinate system and project our pandemic space onto the Euclidean space. By defining the pandemic space, any similarity in the level of prevalence can be quantified by the distance between countries within the space. In fact, the concept behind pandemic space is similar to the idea of social space that is used in social network analysis, and has various applications in different fields [20,21,22]. In social network analysis, the tightness of the social relationship between two individuals is assessed by measuring the distance between them in a social space. Typically, the locations of the individuals in a social space are latent variables and have to be estimated using data. Similarly, in our pandemic network analysis, we study the effect of COVID-19 related policies by tracking the distance between countries in a pandemic space.

With reference to the classical SIR model [23], we may describe the manner in which a pandemic evolves as moving through four stages. In stage zero, there are no confirmed cases, or occasionally there might be a few cases in a country. At this stage, the contribution to the pandemic risk is low. Then, during the first stage, the daily number of new confirmed cases increases and accelerates, indicating the beginning of rapid transmission and an increase in the pandemic risk. The second stage begins some time after the initial outbreak, and is characterized by the imposition of control measures against the outbreak and a deceleration in the daily number of new confirmed cases, which peaks during this stage. It is at this stage that the contribution to the pandemic risk is at its highest. Finally, in the third stage, the daily number of new confirmed cases drops. During this stage, the contribution to the pandemic risk is decreasing. As shown in Appendix E, one characteristic between stages is that the correlation of the daily number of new confirmed cases between countries is low when their contributions to the pandemic risk are in different stages. Therefore, when a country has adopted a better policy to control the spread of the disease, that country will enter the next stage faster than other countries. Even if a country has reached a later stage, a loophole in the preventive measure can lead to another wave of rapid transmission.

To express stochastically the effect on the pandemic risk of the stage at which a country finds itself within the pandemic space, we constructed dynamic pandemic networks that link pairs of countries based on whether those two countries are highly correlated. We did this because we knew that those countries that are in the same stage, except when both are in stage zero, are highly correlated. Given a snapshot of the dynamic network at any particular time point, a situation of high connectedness implies that there is similar prevalence, either an upward, a flattened, or a downward trend in the pandemic risk, in most countries. We expect that in a situation like this, there may occur major events related to COVID-19 and that these events may happen either across a group of several countries or right across the whole world. These major events might include, but are not limited to, rapid transmission of the disease across countries at a time when people remain unaware of its infectiousness and severity, simultaneous lockdown restrictions and border closures across a group of countries, and large-scale vaccination. Otherwise, we should see multiple clusters or groups, which would be indicative of a situation in which there are different levels of prevalence or different stages of pandemic evolution in different countries.

By taking this pandemic space perspective, it is possible to detect similarities in the prevalence of each country in contributing to the pandemic risk by measuring the distance between two countries in the pandemic space. When two countries get closer in the pandemic space, we expect that they will exhibit similar patterns of infections (or a higher probability of being linked together in the pandemic network) and that there will be a similar level of prevalence between these two countries. While by using dynamic pandemic networks, it is possible to detect occasional fluctuations in the linkages between two countries at consecutive time points, we can use the pandemic space to detect fluctuations more accurately and robustly based on the distance between two countries over time. Our model can also estimate which countries exhibit similarities in cases where they may not be linked through the pandemic networks.

There are several ways to measure the distances between countries in the pandemic space. One possible approach is to make use of the network statistics in pandemic network data [24]. There have been recent research papers published that propose the use of COVID-19 pandemic network data to predict and estimate the pandemic risk across countries [25,26,27,28]. The authors of those papers based their conclusions on the study of pandemic risk scores and network connectedness, using network density, the clustering coefficient, and the assortativity coefficient. These statistics are useful for tracking pandemic risk and can also offer an early indication of an acceleration in the number of confirmed cases. Our main contribution in this paper is to construct the pandemic space using a latent pandemic space modeling approach, which provides a view of the pandemic network data that is different from that provided by an analysis that relies on using network statistics. We first performed latent pandemic space modeling [29] using the pandemic network data to determine a time-dependent location for each country, and then measured the distance between every pair of countries via their coordinates in the pandemic space. This practice of applying latent space modeling to analyze network data can be dated back to the use of multidimensional scaling on sociometric data [30]. Since then, there have been further advancements in the analysis of social network data of both static [31] and dynamic [21] networks. Our proposed pandemic space concept, together with the latent space modeling, enables us to visualize clusters of countries representing different levels or stages of pandemic risk contribution at any time point and keep tracking the risk over time. Furthermore, this latent pandemic space model can estimate the size of an effect on the risk of pandemic based on distance in the pandemic space, and the country-specific effect of the distance on the probability of being linked.

The remainder of the paper proceeds as follows. Section 2 describes the statistical methods that we have used to construct the pandemic space. Section 3 gives the results of our analysis, while Section 4 provides a discussion of results. Finally, Section 5 offers some conclusions.

## 2. Materials and Methods

### 2.1. Construction of Pandemic Network

As mentioned in the introduction, we built the pandemic space from dynamic pandemic networks using latent pandemic space modeling. The dynamic pandemic networks consisted of 164 countries, visualized as nodes in the networks. We list the details of the 164 countries, including the total infected numbers in those countries as of 22 July 2020, in Appendix D. The edges among the countries in the pandemic networks change every day in our study period. With reference to the number of confirmed cases in the WHO’s situation reports [32], we linked a pair of countries by an edge in day *t* if, for the last 14 days (i.e., from day t−13 to day *t*), the correlation between the daily change in square root of the cumulative number of cases was larger than 0.5 [25,26,27]. The study period was from 21 January 2020 to 22 July 2020. Following this [16,25], we made use of a moving-window scheme to calculate the 14-day historical correlation. We constructed the network starting from the fourteenth day onward. In the statistical estimation, we regarded the fourteenth day in the data (i.e., 4 February 2020) as day 1 in our network. The data were sufficient for us to create time-varying pandemic networks of T=170 days.

Denote $C_{it}$ as the number of confirmed cases of COVID-19 in country *i* on day *t*, where i=1,…,n, and t=−12,…,T. We performed square root transformation to the number of cases in country *i* from day t−1 to day *t* before calculating the daily increment of cases, i.e.,

(1) $$D_{it}=\sqrt{C_{it}}-\sqrt{C_{i(t-1)}},$$

to make the counts more stable statistically [26,27,33]. Then, to determine the growth pattern between country *i* and *j*, we computed the 14-day historical correlation between $D_{it}$ and $D_{jt}$ for i≠j, i,j=1,…,n, and t=1,…,T, i.e.,

(2) $$\rho_{ijt}=\frac{\sum_{k=1}^{14}\left(D_{i(t-k+1)}-{\bar{D}}_{it}\right)\left(D_{j(t-k+1)}-{\bar{D}}_{jt}\right)}{\sqrt{\sum_{k=1}^{14}{\left(D_{i(t-k+1)}-{\bar{D}}_{it}\right)}^{2}}\sqrt{\sum_{k=1}^{14}{\left(D_{j(t-k+1)}-{\bar{D}}_{jt}\right)}^{2}}},$$

where ${\bar{D}}_{it}=\sum_{k=1}^{14}D_{i(t-k+1)}$ is the 14-day moving average. Finally, for the pandemic network at day *t*, we assigned the (i,j) entry of the adjacency matrix $Y_{t}$ to be 1 if the historical correlation $\rho_{ijt}$ was greater than 0.5 [27], and 0 otherwise. The collection of all adjacency matrices formed the dynamic network of pandemic Y. In case when the correlation was undefined, we followed the approach in Appendix A to determine the corresponding value in $Y_{t}$.

### 2.2. Pandemic Space via Latent Space Modeling

A novelty of the latent space modeling approach is its ability to construct a pandemic space from which we can study the tightness between the pandemic situations in different countries over time graphically. Assume that each country has a latent position on the *s*-dimensional pandemic space, where s=2 in our case for the sake of visualization. Each country moves in the pandemic space every day to reflect the change in pandemic risk. If two countries are close to each other, the probability that they will have co-movement in their number of confirmed cases (i.e., be linked in the network) is high. In the pandemic perspective, this co-movement in the number of confirmed cases can be partly attributed to the possibility of cross-border transmission in the early stages of the pandemic, and similarities in the effect of pandemic preparedness between the two countries—infection measures and hygiene awareness for stopping local and community transmission, and vaccination in the future.

Let $Z_{t}$ be an n×s matrix so that its *i*-th row $Z_{it}$ is the position of country *i* at day *t* on the pandemic space. We assume the initial coordinates $Z_{1}$ follow a multivariate normal distribution, with the joint density

$$\pi \left(Z_{1}|\tau^{2}\right):=\prod_{i=1}^{n}N\left(Z_{i1}|0,\tau^{2}I_{s}\right).$$

Furthermore, we assume that the transition of a latent position in the pandemic space from t−1 to *t* is also normally distributed, with the joint density

$$\pi \left(Z_{t}|Z_{t-1},\sigma^{2}\right):=\prod_{i=1}^{n}N\left(Z_{it}|Z_{i(t-1)},\sigma^{2}I_{s}\right),$$

for t=2,3,…,T, where $I_{s}$ is the s×s identity matrix and N(z|μ,Σ) is the (multivariate) normal density evaluated at z with mean μ and covariance matrix Σ. The parameter $\tau^{2}$ is involved in the calculation of the average initial distance, $E||Z_{i1}-Z_{j1}\left|\right|$, in the pandemic space between countries on day 1. Since the distance $\left|\right|Z_{i1}-Z_{j1}\left|\right|$ between country *i* and *j* is $\sqrt{2}\tau \sqrt{\chi_{2}^{2}}$ distributed and has a mean of $\tau \sqrt{\pi }$, we can use the mean distance to estimate the pandemic risk across countries at t=1. The parameter $\sigma^{2}$ is involved in specifying the distribution of the daily transition in the latent position, $\left|\right|Z_{it}-Z_{i(t-1)}\left|\right|$. It can be shown that $\left|\right|Z_{it}-Z_{i(t-1)}\left|\right|$ is distributed as $\sigma \sqrt{\chi_{2}^{2}}$ and has a mean of $\sigma \sqrt{\pi /2}$. This mean helps us to understand how fast a country contribution of pandemic risk can build up or fall off due to the changes in latent positions. If this mean is large, we expect that two countries separated by long distance on the pandemic space might get closer over a short period of time.

To connect the latent position to the pandemic network, we first re-write the joint density of the adjacency matrix $Y_{t}$ at day *t* to be

$$\begin{array}{cc} P(Y_{t}|Z_{t})= & \prod_{i<j}P{\left(y_{ijt}=1|Z_{t}\right)}^{y_{ijt}}P{\left(y_{ijt}=0|Z_{t}\right)}^{1-y_{ijt}}\\ = & \prod_{i<j}p\left(y_{ijt}\right), \end{array}$$

where

$$p\left(y_{ijt}\right)=\frac{\exp(y_{ijt}\eta_{ijt})}{1+\exp(\eta_{ijt})}$$

also depends on $\eta_{ijt}=\log P\left(y_{ijt}=1|Z_{t}\right)-\log P\left(y_{ijt}=0|Z_{t}\right).$ Then, we model $\eta_{ijt}$, the logit of the conditional probability, with

(3) $$\begin{array}{cc} \eta_{ijt} & =\beta \left(2-d_{ijt}\left(\frac{1}{r_{i}}+\frac{1}{r_{j}}\right)\right), \end{array}$$

where the parameters carry the following meaning:

- $d_{ijt}=∥Z_{it}-Z_{jt}∥$, the distance between two countries in the pandemic space;
- β>0, the overall effect of distance on the link probability $P(y_{ijt})$ and the associated pandemic risk;
- $r_{i}>0$ (with constraint $\sum_{i}r_{i}=1$), can be interpreted as the country-specific effect of the distance on the link probability.

The parameters $r_{i}$ and $r_{j}$ are two factors based on country *i* and *j* respectively to adjust the effect of the distance $d_{ijt}$ on the logit of the link probability $\eta_{ijt}$. Comparing two pairs of countries, *i* and *j*, and $i^{\prime }$ and *j*, if the ratio of the country-specific risk factors of country *i* to country $i^{\prime }$ is the same as the ratio between country *j* to country $j^{\prime }$, i.e., $r_{i}/r_{i^{\prime }}=r_{j}/r_{j^{\prime }}=k$ for some k>1, these two pairs have the same probability of being linked when $d_{ijt}=kd_{i^{\prime }j^{\prime }t}$. In other words, even though the distance between country *i* and *j* is *k* times of the distance between country $i^{\prime }$ and $j^{\prime }$, their high country-specific risk factors counterbalance the distancing effect, leading to the same link probability, which implies a similar level of prevalence risk between country *i* and *j* and between country $i^{\prime }$ and $j^{\prime }$. An application of the country-specific risk is that when the distance between country *i* and *j* is equal to the harmonic mean of their corresponding country-specific risk, i.e., $d_{ijt}=2/\left(r_{i}^{-1}+r_{j}^{-1}\right)$, these two countries have a probability 0.5 of being linked together. Based on $r_{i}$ and $r_{j}$, it is useful to determine a cut-off distance to classify countries *i* and *j* into a cluster, or a group, based on the possibility of their being in the same stage in contributing to the pandemic risk. With the latent space location of country *i* as the center and $r_{i}$ as the radius, we can draw a circle around country *i*. Graphically, we can determine whether a pair of countries, say country *i* and *j*, have a probability of being linked together exceeding 0.5 by considering whether their pandemic space locations lie inside the circles for country *i* and *j* simultaneously.

Parameter β works like a regression coefficient to measure the effect of the distance $d_{ijt}$ on the logit link $\eta_{ijt}$. It also determines the maximum probability for two countries being linked together. The maximum is attained when two countries coincide in the pandemic space, that is, when $d_{ijt}=0$. In this case, the conditional probability is exp(2β)/(1+exp(2β)), which is strictly increasing and reaches 1 when β→∞. Therefore, in the pandemic space, even if two countries are separated by a distance of zero, this does not imply that there must be a link between them, as β is finite.

One remark on the position of countries on the pandemic space is that any distance-preserving transformation in the pandemic space gives an identical value of $\eta_{ijt}$. We followed the approach in Appendix B to handle the identifiability issue.

### 2.3. Estimation of Parameters

We adopted Bayesian methods for estimating the unknown parameters $\tau^{2}$, $\sigma^{2}$, $r_{i}$, and β in the latent space model. First of all, we assigned a normal prior for β, an inverse gamma prior for $\tau^{2}$ and $\sigma^{2}$, and a Dirichlet prior for *r*. All the priors were set to be uninformative to allow variability. However, the full posterior is highly complex and intractable. Therefore, we performed Markov chain Monte Carlo (MCMC) sampling with a total of 200,000 iterates to obtain the posterior estimate. This approach has been widely adopted in many previous Bayesian analyses [29,34,35,36,37,38,39,40]. All full conditional densities required for MCMC are listed in Appendix C.

Denote $\zeta^{\left(l\right)}$ as the parameter ζ, which is one of the parameters to be estimated in the *l*-th iteration, and ${\hat{\zeta }}^{\left(l\right)}$ as the proposed value in the *l*-th iteration, which was randomly drawn from the proposal distribution with density $f_{\zeta }\left(\cdot ;\theta_{\zeta }\right)$, where $\theta_{\zeta }$ governs the step size of the proposal. At the beginning of Markov chain Monte Carlo (MCMC), we set an initial value $\zeta^{\left(0\right)}$ for each parameter. We also set this value to be the prior mean. In each iteration, we sequentially sampled all unknown parameters from proposal distribution. In particular, during the l+1-th iteration, we

1. Draw β from $N(\beta^{\left(l\right)},\theta_{\beta })$ with truncation on the non-positive values.
2. Draw $\log\tau^{2}$ from $N(\log\tau^{2^{\left(l\right)}},\theta_{\log\tau^{2}})$.
3. Draw $\log\sigma^{2}$ from $N(\log\sigma^{2^{\left(l\right)}},\theta_{\log\sigma^{2}})$.
4. Draw *r* from Dirichlet distribution with concentration parameter $\theta_{r}r^{\left(l\right)}$.
5. Draw $Z_{it}$ from $N(Z_{it}^{\left(l\right)},\theta_{Z_{it}}I_{s})$, for i=1,…,n, and t=1,…,T.

In each of the above steps, we calculate the acceptance probability

(4) $$\alpha \left({\hat{\zeta }}^{\left(l+1\right)},\zeta^{\left(l\right)}\right)=\min\left\{1,\frac{\pi \left({\hat{\zeta }}^{\left(l+1\right)}\right)f_{\zeta }\left(\zeta^{\left(l\right)}\right)}{\pi \left(\zeta^{\left(l\right)}\right)f_{\zeta }\left({\hat{\zeta }}^{\left(l+1\right)}\right)}\right\},$$

where π(ζ) is the posterior density of ζ, to decide whether to accept the proposed value ${\hat{\zeta }}^{\left(l+1\right)}$ as the next current value, i.e., setting $\zeta^{\left(l+1\right)}={\hat{\zeta }}^{\left(l+1\right)}$. If not, we reject the proposed value and keep the current value, i.e., setting $\zeta^{\left(l+1\right)}=\zeta^{\left(l\right)}$.

**Figure 1:**
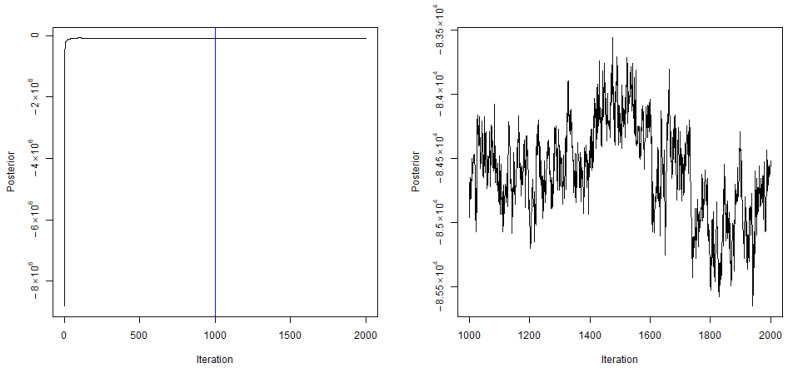
Posterior (**left**) when the observation was drawn. The plot on the **right**-hand side focuses on the last 50% of observations after the burn-in.

We also implemented the following MCMC adaptations to improve the convergence of results [41,42]. In the first stage, which consists of the first 5% of iterates, we allowed the Markov chain to move in order to find the best initial position. In the second stage, we controlled the acceptance rate by adjusting the step size (i.e. $\theta_{\zeta }$) until we completed the first 30% of iterates. After the MCMC adaptations, we let the chain burn-in by neither changing the step size nor including them into our sample for the next 20% of iterates. Finally, after the burn-in period, we took the last 50% of iterates to estimate the posterior. In order to reduce the autocorrelation and speed up the convergence, we recorded one observation for every 100 iterations. Moreover, starting from the 10% of total iterates, every time after we recorded an observation, we calculated the effective sample size, which was measured by the reciprocal of autocorrelation in the latest 100 iterates, and used it to determine the probability for each parameter to be sampled in the next iteration. This probability was fixed after we completed 30% of total iterates. In Figure 1, we provide a plot of the posterior to show the convergence. The plot on the left-hand side shows the posterior in each MCMC iteration. The plot on the right-hand side focuses on the post-burn-in period, with which our posterior estimates and standard deviation were calculated.

## 3. Results

In this section, we present the statistical results from the latent pandemic space model. As mentioned in previous sections, we linked two countries together and believed that they had a similar level of prevalence if the growth pattern of their confirmed COVID-19 cases showed a certain extent of similarity. Using a 14-day moving-window scheme allowed us to gather enough observations to calculate moving correlations, and also reduce fluctuation due to a single-day spike in the data. The WHO’s situation report records the daily number of confirmed cases since 21 January 2020. As we needed 14 days data to calculate the correlation, the study period of our pandemic network was from 4 February 2020 to 22 July 2020.

**Figure 2:**
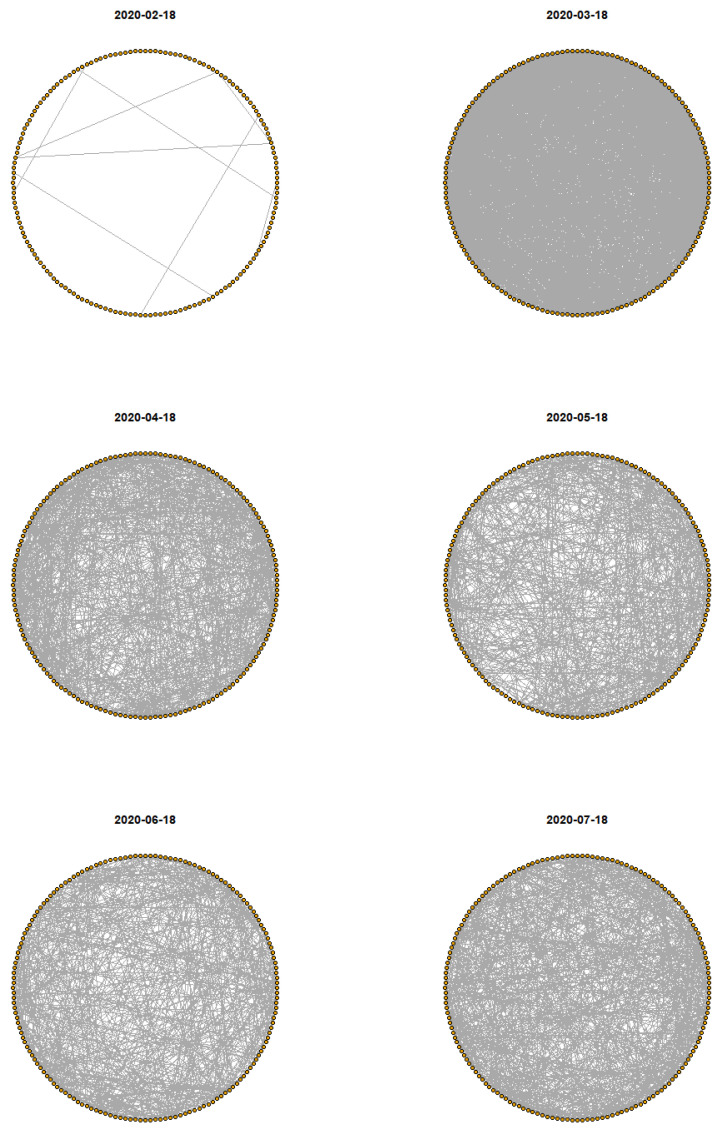
Snapshots of the pandemic network on the eighteenth day of each month from February to July 2020.

Before analyzing the modeling results, we followed [25] to present Figure 2, which shows the pandemic network on the eighteenth day of each month. We can see that in February, there are only a few edges. One month later, we see that there are many more edges in the pandemic network, indicating that more countries are experiencing similar levels of prevalence. If we also take into account the daily number of confirmed cases at that time, it begins to appear that there were major events that accelerated the pandemic risk during that period. After that, although the number of edges in April to June was fewer than in March, indicating a diverging scenario in the daily number of confirmed cases, most countries had at least one link to another country, showing that the pandemic risk (between countries, or community transmission) may be lower but had not disappeared. For July, we again found an increment in the number of edges. We believe that this should be interpreted as signaling an upcoming change in the pandemic risk. This signal can also be detected in the preparedness risk score [25].

**Figure 3:**
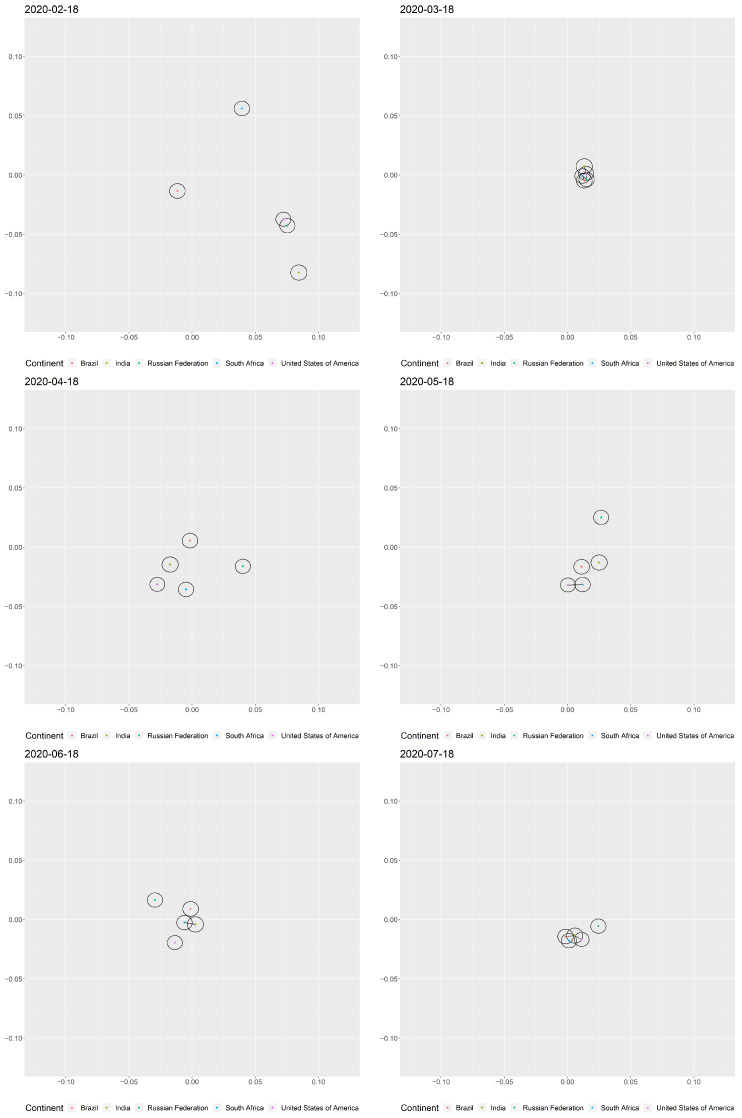
Snapshots of the latent positions of those countries that were in the top 5 list in terms of the cumulative number of confirmed COVID-19 cases on the eighteenth day of each month from February to July 2020. Inclusion of centers in two circles implies that the corresponding pair of countries had a similar level of prevalence, either because of possible transmission between the countries or because there was a common trend in the severity of community transmission. The line segment between two countries refers to a link between them on that day in the pandemic network. To view an animation of the changes in the latent positions in the pandemic space, please go to “Top 5 list” (https://drive.google.com/file/d/18ENvRXQlvaWJFSyBregzB_sLQ3Ewm7P7/view?usp=sharing), or “All countries” (https://drive.google.com/file/d/1a0oWJJm47N9cQEsg02_RhpEWIchQu56n/view?usp=sharing).

In our estimated pandemic space, we can visualize the above changes in network connectedness in terms of the distances between countries. A shorter distance implies a higher probability of connection and thus a synchronized change in the prevalence of cross-border or community transmission in two countries. Figure 3 shows the pandemic space on the eighteenth day in each month with the top five countries having the highest number of confirmed cases of COVID-19. As of 22 July 2020, the five countries which contributed around 60% of the total number of infections were the United States of America, Brazil, India, the Russian Federation, and South Africa [2]. In Figure 3, the radii of those circles surrounding the country names represent the country-specific effects. Depending on whether the distance between two countries is smaller than or larger than both radii of their corresponding circles, we can determine whether the probability of a link between them is more than or less than 50%. In particular, if two countries have the same country-specific effect, and if the center of one circle lies on the boundary of the second circle, and vise versa, the probability of the two countries being linked is exactly 50%. Since all circles have similar radii in our case, we can simply consider whether their centers are included in both circles in order to classify whether or not there is a similar level of prevalence between two countries. Therefore, excluding those marginal cases, among the top five countries in the figure, USA–India, USA–South Africa, USA–Russia, Brazil–Russia, Brazil–South Africa, and Russia–South Africa had potentially similar levels of prevalence on 18 March 2020; and USA–India, India–South Africa, and Brazil–South Africa had potentially similar levels of prevalence on 18 July 2020.

**Figure 4:**
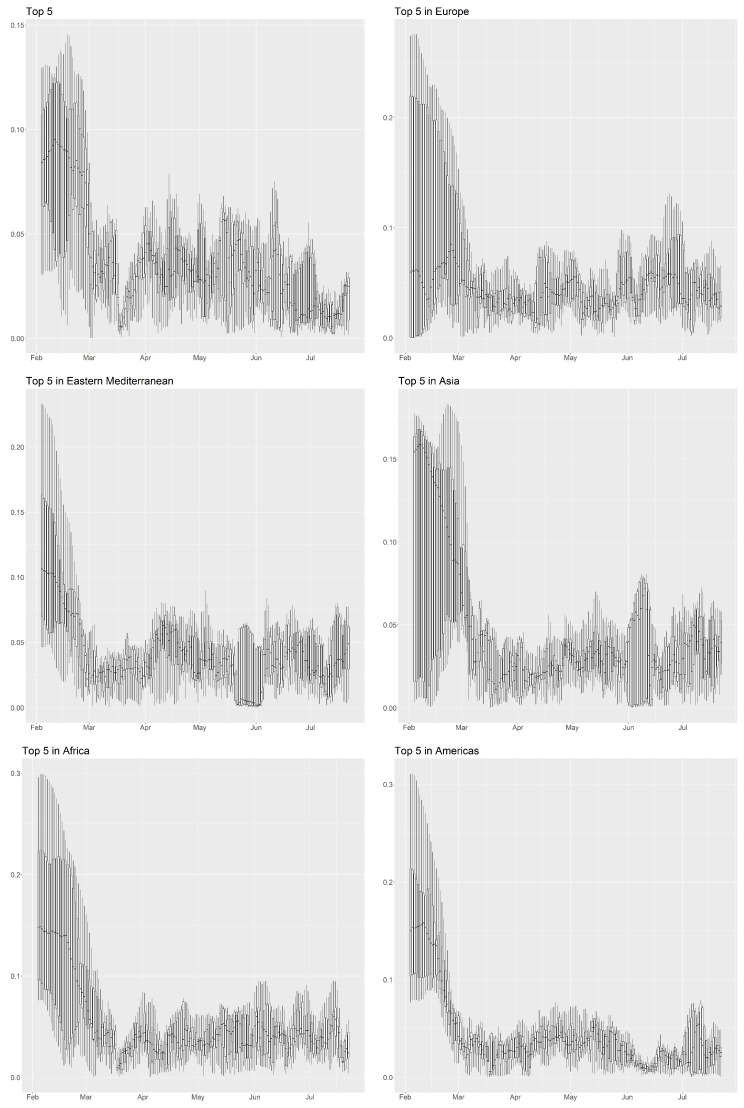
Time series of the boxplots of the distance among countries, which are in the top 5 in terms of the cumulative number of confirmed COVID-19 cases in the whole world or in each of the five continents.

Figure 4 studies the pandemic risk between countries. We again selected those countries which were in the top five list in terms of the cumulative number of confirmed COVID-19 cases and produced boxplots of the distance between each pair of countries over time. To construct the boxplots, we calculated the distance according to the latent coordinates in the pandemic space, a total of 10 distances from the top five countries in each day. In Figure 4, we categorize the countries by continent according to the WHO’s classification, which has also been implemented in the literature on pandemic network research [25]. We created daily boxplots for the five regions—Africa, the Americas, Asia, the Eastern Mediterranean, and Europe—and we aggregated the top five countries in each of the five regions and labeled them as “Top 5”. In general, it is unlikely that all countries would be separated by small distances, as this would imply a high degree of similarity in prevalence between all countries. However, if this is the case, we will see that the boxplots are short and located at a low position. Based on the variability and maximum distances, we have a conservative measure of classifying the periods of high pandemic risk based on the network connectedness. From the “Top 5" graph for the whole world, the pandemic emerges in February 2020, when the median distances are large. The situation seems to get worse in late February 2020, when the median distance drops more than 50% in a week. In fact, the highest levels of connectedness are mid-March and late June to mid-July 2020, at the times of the two main waves of the pandemic. In addition to the two periods previously identified, there are also two other periods of high connectedness or potentially high pandemic risk in the five regions: one in Europe during mid-May 2020, and the other in the Americas during mid-June 2020.

**Figure 5:**
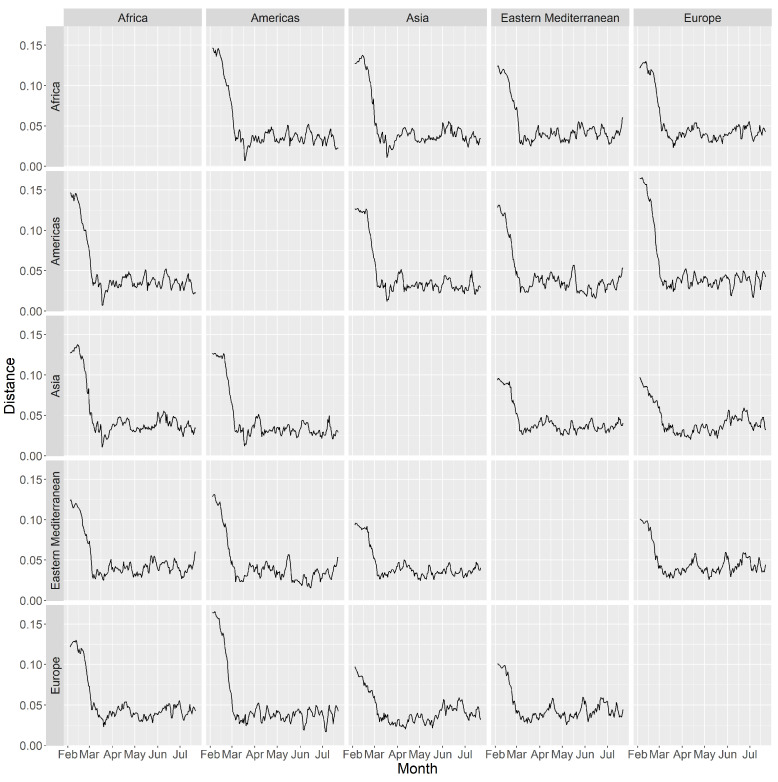
Time series plot of the median distance between clusters of the top five countries from each continent.

Besides intra-continental pandemic risk, we also studied inter-continental risk. In Figure 5, we calculated for each day the median distances between clusters of the top five countries from each continent. For example, if country A belongs to Africa, and country B belongs to the Americas, we include the distance between A and B when we calculate the median for the Africa-Americas distance (i.e., the second plot in the first row of the matrix plot in Figure 5). We observe that the distances in all plots decrease from around 0.125 in late February to 0.025 in mid-March, showing that the “inter-continental risk” builds up quickly in this period. The distances stay in the range of 0.025 to 0.05 for most of the time after March. For the plots among Africa, the Americas, and Asia, the minimum distance over the whole period of study is attained in mid-March. The distance between Europe and the Americas is relatively small in early June and early July. From Figure 5, we cannot observe any sign that the pandemic is coming to an end as the distances are mostly below 0.05 till mid-July 2020.

Table 1 summarizes the results with the estimate (posterior mean) and the standard deviation (SD) of each parameter in our pandemic space model. The parameter β determines the highest probability of linking two countries when they have the same location, or $d_{ijt}=0$. Based on the estimate of β, the highest probability was 72.40%. The parameter τ and σ are the standard deviations of latent coordinates on the first day, and of the daily transition in coordinates, respectively. Therefore, in this pandemic space, we can estimate the mean distance between countries on the first day by $\tau \sqrt{\pi }=0.1504$, and the average distance each country travels in one day by $\sigma \sqrt{\pi /2}=0.0100$. Consistent with the results shown in Figure 4, the initial pandemic risk between countries is small. Potentially high pandemic risk (i.e., small distance) can be seen in two weeks from mid-February, with reference to Figure 5, such that the median of distances is not greater than 0.05 for most of the time. From the latent space modeling, we can deduce that the COVID-19 pandemic evolves quickly and that the outbreak situation can become worse within a matter of just two weeks.

The variable invr is the mean of the inverse of country-specific risk factor, $r_{i}^{-1}$. Based on our model assumptions, the sum of all country-specific risk factors is fixed to be 1 for the sake of identifiability. If invr attains its minimum value, which is the number of countries, every country has the same probability of linking to another country provided that their distances are the same. On the other hand, countries having larger $r_{i}$ will have a relatively higher probability of linking to another country. In this study, the estimate of invr is close to the minimum, implying that there is no country dominating the contribution to the pandemic risk. From another perspective, if we compare the country-specific risk factor $r_{i}$, Serbia and New Zealand have the minimum and maximum country-specific risk at 0.0057 and 0.0066, respectively. The minimum and maximum are not far from the mean value of country-specific risk of 0.0061. We list the country-specific risk factor for each of the 164 countries in Appendix D.

## 4. Discussion

Although the exact date depends on each country, most countries set up their travel restriction policies by 31 March 2020 [43]. Therefore, we set 31 March 2020 as a cutoff date for further discussion. Prior to travel restrictions coming into effect, a traveler who was an asymptomatic COVID-19 carrier could transmit the disease to other people in different countries. After the cutoff date, most incoming air traffic was prohibited and many lockdown policies were put in place. The main focus switched to the question of when a suitable time to reopen would be, and another focus was which countries had relatively smaller contributions to the pandemic risk.

In Figure 3, we notice that for 18 March 2020, the first period of increasing pandemic risk observed in Figure 4, there were six pairs of countries out of a total of 10 that had similar levels of prevalence. This result coheres with the rapid increase in the number of edges in the pandemic network. In fact, referring to the literature [27,44], the number of confirmed cases and the network connectedness accelerated during this period. We believe that the importation and exportation risk of COVID-19 cases via air travel poses a risk in the transmission of COVID-19 [45]. Within one month after the declaration of the COVID-19 pandemic, by which time most countries had imposed travel restriction, we see from Figure 3, Figure 4 and Figure 5 the effectiveness of such measures in increasing the distance between countries in the pandemic space compared to the situation in March 2020.

Although none of the 10 country pairs had similar levels of prevalence until mid-July 2020, the distances in the 10 country pairs were not as large as they appear to have been in February 2020. The pandemic risk still existed and had the potential to lead to another wave of rapid transmission if travel restrictions were lifted [46]. We also see that on 18 July 2020, there was one edge between USA and India, and one edge between India and South Africa. The close distances between them put those countries into the cluster of similar prevalence, together with the Brazil–South Africa pair, which had no link on that day. Instead of importation and exportation, we believe that the reason behind this closing distance was the partial re-opening of the two countries’ economies [47] and the permitting of inter-state air travel [48]. In fact, these measures may also be considered the reason behind the period of increased risk in Europe during mid-May 2020 and in the Americas during mid-June 2020. Figure 5 provides another view from the inter-continental perspective, showing that while most of the restrictions on international air travel remained valid, the inter-continental distances in July 2020 stayed in the low-value range (below 0.05), implying that the similarity in the levels of prevalence across continents was still quite high in mid-July 2020.

To investigate the effectiveness of lockdown policy, we refer to the “Top 5 in Europe” plot in Figure 4, as most of the European countries experienced a lockdown in late March to late April 2020. Between mid-March and late March 2020, the median distance stayed small and the boxplots are short, indicating that the impact from the first wave of transmission lasted for at least half a month before the lockdown. After two weeks of lockdown (i.e., by around mid-April 2020) the median distance increased to a relatively high level, and for the same time period in the graph, the boxplots become taller. Similarity in the levels of prevalence between countries is weakened as the implementation of social distancing measures restricts opportunities for transmission of the disease, though it should be noted that the effectiveness of measures against the transmission of COVID-19 varies across countries. We also discovered that the median distances of the boxplot dropped again after early May 2020. However, we found that the daily number of new cases was decreasing at that time, indicating that they had a decreasing contribution to pandemic risk. Around that period of time, most European countries began to re-open in a stepwise manner [49,50]. We believe that the imposition of a lockdown is effective in reducing the pandemic risk, as the distances among countries during periods of lockdown were larger than those before the lockdown in the pandemic space. However, by beginning the process of reopening at a time when the daily number of new cases has not yet reached a stable low value, the distances among countries may decrease again, together with further waves of mass transmission.

## 5. Conclusions

In this paper, we developed the pandemic space approach, which was built upon the concept of dynamic latent space modeling, to explore the pandemic risk across countries around the world. Using the pandemic space, we investigated and provided hints on whether the various control measures adopted in different countries, including travel restrictions and lockdowns, were effective at reducing the pandemic risk or not. We first constructed the pandemic network using 14-day data in a moving window scheme. We linked two countries together if their correlation in terms of numbers of infections was high. We followed this rule to build a daily network from 4 February 2020 to 22 July 2020. Then, based on the pandemic network, we performed latent space modeling to produce our pandemic space.

The pandemic space allowed us to identify country pairs that had potentially high transmission risks in some periods, or a synchronized increase in the severity of community transmission. We also explained the different pandemic periods using the maximum distances and the height from time series boxplots of distances. We also investigated the inter-continental risk with the time series of the median distance between each pair of continents. Moreover, using the parameters obtained from our estimation, we examined the highest probability of two countries being linked, the country-specific effect, the initial average distance, and the speed to build up the pandemic risk. As in other papers [28,51,52,53], using this pandemic space analysis, we concluded that both lockdown and travel restrictions are effective at reducing the pandemic risk across countries. Nevertheless, these two measures, and probably also other control measures, are not sufficient to wipe out the risk of pandemic across countries.

Future works might include considerations of exogenous variables, such as statistics related to travel between countries, the number of people who have received at least one vaccine dose, and lockdown activities, so as to make it possible to conduct Bayesian predictions about similarities in levels of prevalence between countries and the pandemic risk. It would also be interesting to consider pandemic spaces with higher dimensions and interpretations of the latent dimensions in the pandemic space in future research.

## Figures and Tables

**Table 1:** Estimate of the parameters in the latent space model. The first column contains the posterior mean. The second column contains the posterior standard deviation. β is the regression coefficient, τ is the standard deviation of latent coordinates on the first day, σ is the standard deviation of daily transition in coordinates, and invr is the mean of inverse of country-specific factor.

	Estimate	SD
β	0.482092	0.005071
τ	0.084863	0.004512
σ	0.007961	0.000055
invr	164.198509	0.014353

**Figure A1:**
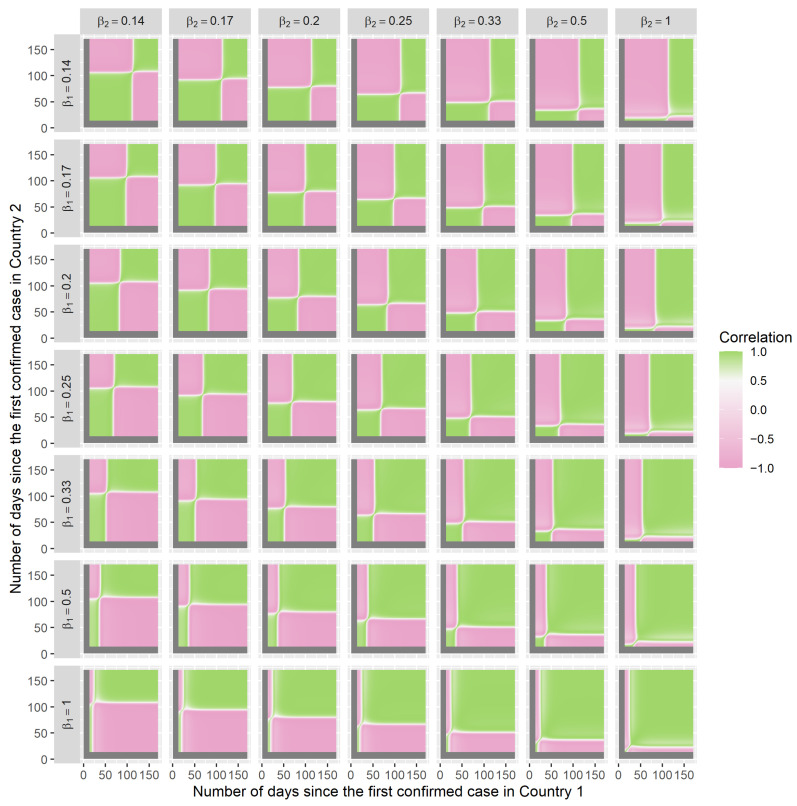
The *x*-axis and *y*-axis in each plot refer to the number of days since the first confirmed case in each of the two countries, respectively. The region in green represents the scenarios when the correlation between the two countries is greater than 0.5.

## Data Availability

The dataset used in this study can be found on https://covid19.who.int/table.

## Abbreviations

The following abbreviations are used in this paper:

Eastern Med.	Eastern Mediterranean

## Appendix A. Handling Missing Values

To allow every country to have a location in the pandemic space on each day, we impute those missing links between countries as empty; i.e., $y_{ijt}=0$. In the pandemic network, a pair of countries has a missing link when the correlation at time *t* is undefined—i.e., there have been no confirmed COVID-19 cases in the previous 14 days for at least one country in that pair. It is reasonable to claim that the pandemic risk between these countries is negligible when a country has had no new cases in 14 consecutive days.

## Appendix B. Identifiability of Latent Position

Since the $\eta_{ijt}$ in Equation (3) depends on the distance between countries in the pandemic space instead of their coordinates, if we hold other variables unchanged, any distance preserving transformation in the pandemic space, like translation, reflection and rotation, gives an identical value of $\eta_{ijt}$. To handle the identifiability issue on latent positions, we use the Procrustes transformation [54] to translate and rotate the whole space at time *t* such that the sum of squared distance traveled by all countries from t−1 to *t*, and from *t* to t+1 is minimized; i.e.,

(A1) $$\min_{Z_{t}}\sum_{i=1}^{n}{∥Z_{it}-Z_{i(t-1)}∥}^{2}+{∥Z_{i(t+1)}-Z_{it}∥}^{2}.$$

## Appendix C. Posterior Distribution

We denote ϕ as all parameters, including β, $\tau^{2}$, $\sigma^{2}$, $r_{i}$, and $Z_{it}$, which are not specified as arguments but appearing in the following posterior density function. We first write the likelihood of Y, as a product of conditional distribution:

$$P\left(Y|\phi \right)=\prod_{t=1}^{T}P\left(Y_{t}|\phi \right)=\prod_{t=1}^{T}\prod_{i<j}p\left(y_{ijt}\right).$$

Then, we derive the following posterior densities:

- The joint posterior density of $Z_{it}$ is
  $$\begin{array}{c} \pi \left(Z_{it}|Y,\phi \right)\propto \left(\prod_{j:j\neq i}p\left(y_{ijt}\right)\right)\cdot \prod_{t^{\prime }=t}^{t+1}N\left(Z_{it^{\prime }}|Z_{i(t^{\prime }-1)},\sigma_{\left(t^{\prime }\right)}^{2}I_{s}\right), \end{array}$$

for t=1,…,T, where $Z_{i0}$ is a zero vector of length *s*, $Z_{i(T+1)}=Z_{iT}$, and

$$\sigma_{\left(t\right)}^{2}=\left\{\begin{array}{cc} \sigma^{2} & \mathrm{if}\;t=2,\ldots ,T,\\ \tau^{2} & \mathrm{if}\;t=1,\\ 0 & \mathrm{otherwise}. \end{array}\right.$$

- The posterior densities of $\tau^{2}$ and $\sigma^{2}$ are
  (A2) $$\begin{array}{c} \pi \left(\tau^{2}|Y,\phi \right)=IG\left(\nu_{\tau^{2}}+\frac{1}{2}sn,\xi_{\tau^{2}}+\frac{1}{2}\sum_{i=1}^{n}{∥Z_{i1}∥}^{2}\right) \end{array}$$

and

(A3) $$\begin{array}{c} \pi \left(\sigma^{2}|Y,\phi \right)=IG\left(\nu_{\sigma^{2}}+\frac{1}{2}sn\left(T-1\right),\xi_{\sigma^{2}}+\frac{1}{2}\sum_{i=1}^{n}\sum_{t=2}^{T}{∥Z_{it}-Z_{i(t-1)}∥}^{2}\right) \end{array}$$

respectively, where $\nu_{\tau^{2}}$ and $\nu_{\sigma^{2}}$ are the shape parameters in the inverse gamma prior, and $\xi_{\tau^{2}}$ and $\xi_{\sigma^{2}}$ are the scale parameters of the inverse gamma prior.

- The posterior density of β is
  (A4) $$\begin{array}{c} \pi \left(\beta |Y,\phi \right)\propto \left(\prod_{t=1}^{T}\prod_{i<j}p\left(y_{ijt}\right)\right)\cdot N\left(\beta |\nu_{\beta },\xi_{\beta }\right), \end{array}$$
  where $\nu_{\beta }$ and $\xi_{\beta }$ are the mean and variance respectively of the normal prior.
- The joint posterior density of $r=(r_{1},\ldots ,r_{n})$ is
  (A5) $$\begin{array}{c} \pi \left(r|Y,\phi \right)\propto \left(\prod_{t=1}^{T}\prod_{i<j}p\left(y_{ijt}\right)\right)\cdot \prod_{i=1}^{n}r_{i}^{\alpha_{i}-1}, \end{array}$$
  where $\alpha_{i}$, for i=1,…,n, are the concentration parameters of the Dirichlet prior.

## Appendix D. Table of Countries

**Table A1:** A list of the 164 countries considered in this study, their total number of cases as of 22 July 2020, their corresponding continents, their ranks in their continents, and their estimated country-specific risks. Countries are listed in descending order of the total number of cases.

Country Name	Total Number of Cases	Continent	Rank in Continent	Country-Specific Risk Factor
United States of America	3,805,524	Americas	1	0.0059
Brazil	2,118,646	Americas	2	0.0062
India	1,192,915	Asia	1	0.0064
Russian Federation	789,190	Europe	1	0.0060
South Africa	381,798	Africa	1	0.0061
Peru	357,681	Americas	3	0.0060
Mexico	349,396	Americas	4	0.0061
Chile	334,683	Americas	5	0.0060
The United Kingdom	296,912	Europe	2	0.0062
Iran	278,827	Eastern Med.	1	0.0060
Spain	278,528	Europe	3	0.0059
Pakistan	267,428	Eastern Med.	2	0.0063
Saudi Arabia	255,825	Eastern Med.	3	0.0058
Italy	244,752	Europe	4	0.0060
Turkey	221,500	Europe	5	0.0061
Bangladesh	210,510	Asia	2	0.0060
Colombia	204,005	Americas	6	0.0061
Germany	202,799	Europe	6	0.0061
France	166,511	Europe	7	0.0059
Argentina	130,774	Americas	7	0.0059
Canada	111,124	Americas	8	0.0062
Qatar	107,430	Eastern Med.	4	0.0063
Iraq	97,159	Eastern Med.	5	0.0061
Indonesia	89,869	Asia	3	0.0060
Egypt	89,078	Eastern Med.	6	0.0061
Kazakhstan	76,799	Europe	8	0.0063
Ecuador	76,217	Americas	9	0.0063
Sweden	74,766	Europe	9	0.0061
Philippines	70,764	Asia	4	0.0064
Oman	69,887	Eastern Med.	7	0.0059
Belarus	66,348	Europe	10	0.0065
Belgium	65,093	Europe	11	0.0062
Ukraine	60,995	Europe	12	0.0060
Bolivia	60,991	Americas	10	0.0061
Kuwait	60,434	Eastern Med.	8	0.0063
United Arab Emirates	57,498	Eastern Med.	9	0.0057
Dominican Republic	54,797	Americas	11	0.0060
Panama	54,426	Americas	12	0.0062
Israel	52,431	Europe	13	0.0061
Netherlands	52,073	Europe	14	0.0059
Portugal	48,898	Europe	15	0.0061
Singapore	48,434	Asia	5	0.0061
Poland	40,782	Europe	16	0.0060
Guatemala	40,229	Americas	13	0.0061
Romania	39,133	Europe	17	0.0062
Nigeria	37,801	Africa	2	0.0061
Bahrain	37,316	Eastern Med.	10	0.0061
Afghanistan	35,813	Eastern Med.	11	0.0061
Armenia	35,693	Europe	18	0.0062
Honduras	34,611	Americas	14	0.0061
Switzerland	33,655	Europe	19	0.0060
Kyrgyzstan	29,359	Europe	20	0.0059
Ghana	28,989	Africa	3	0.0062
Azerbaijan	28,242	Europe	21	0.0060
Japan	26,303	Asia	6	0.0057
Ireland	25,802	Europe	22	0.0060
Algeria	24,278	Africa	4	0.0064
Serbia	21,605	Europe	23	0.0057
Republic of Moldova	21,442	Europe	24	0.0059
Austria	19,818	Europe	25	0.0058
Uzbekistan	18,171	Europe	26	0.0061
Nepal	17,994	Asia	7	0.0061
Morocco	17,742	Eastern Med.	12	0.0061
Cameroon	16,522	Africa	5	0.0060
Cote d lvoire	14,531	Africa	6	0.0063
Czechia	14,324	Europe	27	0.0062
Kenya	14,168	Africa	7	0.0061
Republic of Korea	13,879	Asia	8	0.0058
Denmark	13,302	Europe	28	0.0057
Puerto Rico	12,940	Americas	15	0.0062
El Salvador	12,582	Americas	16	0.0065
Australia	12,428	Asia	9	0.0062
Venezuela	12,334	Americas	17	0.0057
Costa Rica	11,534	Americas	18	0.0064
Sudan	11,127	Eastern Med.	13	0.0061
Ethiopia	11,072	Africa	8	0.0062
North Macedonia	9412	Europe	29	0.0061
Bulgaria	9254	Europe	30	0.0059
Norway	9038	Europe	31	0.0065
Senegal	8985	Africa	9	0.0060
Malaysia	8815	Asia	10	0.0064
Bosnia and Herzegovina	8786	Europe	32	0.0062
Democratic Republic of the Congo	8533	Africa	10	0.0063
Finland	7351	Europe	33	0.0063
Guinea	6625	Africa	11	0.0062
Gabon	6433	Africa	12	0.0064
Mauritania	5985	Africa	13	0.0064
Luxembourg	5725	Europe	34	0.0062
Djibouti	5027	Eastern Med.	14	0.0062
Central African Republic	4561	Africa	14	0.0062
Croatia	4422	Europe	35	0.0062
Hungary	4366	Europe	36	0.0060
Albania	4290	Europe	37	0.0060
Greece	4048	Europe	38	0.0062
Paraguay	3748	Americas	19	0.0060
Zambia	3326	Africa	15	0.0060
Thailand	3261	Asia	11	0.0063
Somalia	3135	Eastern Med.	15	0.0062
Maldives	3044	Asia	12	0.0060
Nicaragua	3004	Americas	20	0.0058
Lebanon	2980	Eastern Med.	16	0.0061
Congo	2851	Africa	16	0.0061
Sri Lanka	2730	Asia	13	0.0058
Montenegro	2567	Europe	39	0.0061
Cuba	2449	Americas	21	0.0059
Equatorial Guinea	2350	Africa	17	0.0063
Estonia	2022	Europe	40	0.0060
Slovakia	2021	Europe	41	0.0060
Slovenia	1977	Europe	42	0.0059
Lithuania	1949	Europe	43	0.0060
Eswatini	1894	Africa	18	0.0064
Iceland	1839	Europe	44	0.0058
Benin	1690	Africa	19	0.0061
Rwanda	1655	Africa	20	0.0061
Tunisia	1394	Eastern Med.	17	0.0061
Namibia	1366	Africa	21	0.0065
New Zealand	1205	Asia	14	0.0066
Latvia	1193	Europe	45	0.0062
Jordan	1181	Eastern Med.	18	0.0063
Liberia	1108	Africa	22	0.0062
Niger	1108	Africa	23	0.0061
Suriname	1079	Americas	22	0.0065
Georgia	1073	Europe	46	0.0060
Burkina Faso	1065	Africa	24	0.0062
Uruguay	1064	Americas	23	0.0059
Cyprus	1040	Europe	47	0.0061
Chad	889	Africa	25	0.0060
Andorra	884	Europe	48	0.0063
Jamaica	809	Americas	24	0.0059
Togo	790	Africa	26	0.0066
San Marino	716	Europe	49	0.0060
Malta	675	Europe	50	0.0060
United Republic of Tanzania	509	Africa	27	0.0059
Viet Nam	401	Asia	15	0.0059
Mauritius	343	Africa	28	0.0061
Guyana	337	Americas	25	0.0059
Guam	319	Asia	16	0.0060
United States Virgin Islands	308	Americas	26	0.0063
Mongolia	287	Asia	17	0.0058
Cayman Islands	203	Americas	27	0.0063
Cambodia	197	Asia	18	0.0061
Faroe Islands	191	Europe	51	0.0060
Gibraltar	180	Europe	52	0.0061
Bahamas	174	Americas	28	0.0061
Bermuda	153	Americas	29	0.0062
Brunei Darussalam	141	Asia	19	0.0062
Trinidad and Tobago	137	Americas	30	0.0062
Gambia	132	Africa	29	0.0060
Aruba	115	Americas	31	0.0061
Seychelles	108	Africa	30	0.0060
Barbados	106	Americas	32	0.0059
Bhutan	92	Asia	20	0.0064
Liechtenstein	87	Europe	53	0.0061
Monaco	81	Europe	54	0.0060
Sint Maarten	79	Americas	33	0.0058
Antigua and Barbuda	76	Americas	34	0.0061
French Polynesia	62	Asia	21	0.0062
Saint Vincent and the Grenadines	50	Americas	35	0.0060
Saint Martin	46	Americas	36	0.0062
Curacao	28	Americas	37	0.0061
Fiji	27	Asia	22	0.0060
Saint Lucia	23	Americas	38	0.0059
New Caledonia	22	Asia	23	0.0059
Greenland	13	Europe	55	0.0058

## Appendix E. Effects of Infection Parameters and Recovery Parameters in the SIR Model on Correlations

In this appendix, we demonstrate with heatmaps the 14-day historical correlations of the daily number of new confirmed cases between pairs of countries under the susceptible-infected-recovered (SIR) model. The SIR model assumes that among the population *N* who get infected with a certain kind of disease, people can be divided into three classes. The first class “susceptible”, S(t), contains individuals who are at risk at time *t*. The second class “infected”, I(t), contains those individuals who have been infected and are able to spread the diseases. The third class “recovered”, R(t), contains those who have recovered from the infection and have become immune to the disease. We can use the following mathematical equations to formulate the relationship among these three classes:

(A6) $$\begin{array}{ccc} \frac{dS(t)}{dt} & = & -\frac{\beta S(t)I(t)}{N},\\ \frac{dI(t)}{dt} & = & \frac{\beta S(t)I(t)}{N}-\gamma I\left(t\right),\\ \frac{dR(t)}{dt} & = & \gamma I(t), \end{array}$$

where β is the infection parameter and γ is the recovery parameter. In our demonstration, we assume that the recovery parameter is equal to one-tenth of the infection parameter for simplicity. Moreover, we allow countries 1 and 2 to have infection parameters $\beta_{1}$ and $\beta_{2}$, respectively. We repeat the calculation after changing these two parameters to be combinations of 0.14,0.17,0.2,0.25,0.33,0.5, and 1. Following Equation (1), we first calculate the square root transformed daily number of new cases, $D_{t}=\sqrt{S(t-1)}-\sqrt{S(t)}$. Then, we calculate the correlation by Equation (2) from t=1 to t=170. Notice that in the first 13 days, there are insufficient data to calculate the correlation. Therefore, the corresponding entries are colored in grey. In Figure A1, the *x*-axis and *y*-axis in each plot refer to the number of days since the first confirmed case in the two countries. The region in green represents the scenarios when the correlation between the two countries is greater than 0.5. We can roughly see three stages: one at the bottom left, a bridge in the middle, and one at the top right.
